# Transepidermal Water Loss and T-helper 2 (Th2)-Associated Inflammatory Markers in Two Pediatric Patients During the First Four Weeks of Treatment With the Oral Janus Kinase Inhibitor Upadacitinib

**DOI:** 10.7759/cureus.51196

**Published:** 2023-12-27

**Authors:** Kenta Horimukai, Misako Kinoshita, Noriko Takahata

**Affiliations:** 1 Department of Pediatrics, Jikei University Katsushika Medical Center, Tokyo, JPN

**Keywords:** upadacitinib, transepidermal water loss, tarc, skin barrier function, managing atopic dermatitis, janus kinase (jak) inhibitors

## Abstract

Few studies have evaluated the effects of upadacitinib on skin barrier function and T-helper 2 (Th2)-associated inflammatory biomarkers in severe atopic dermatitis (AD). In this study, we followed two pediatric patients with AD who had previously failed to respond to conventional treatment and measured their serum Th2-associated chemokine thymus and activation-regulated chemokine (TARC) and serine protease inhibitor squamous cell carcinoma antigen (SCCA) 2 levels and transepidermal water loss (TEWL) during the first four weeks of upadacitinib treatment. Both patients showed marked clinical improvement and decreased TEWL, blood eosinophil counts, and serum TARC and SCCA2 levels after four weeks of upadacitinib treatment. These findings suggest that upadacitinib attenuates Th2-associated inflammatory markers and promotes skin barrier integrity.

## Introduction

Atopic dermatitis (AD) is a common chronic skin disease characterized by skin barrier dysfunction and type 2 inflammation [1]. Transepidermal water loss (TEWL) is a key clinical indicator of skin barrier function. For example, in adults, twice-daily topical application of potent topical steroids to the skin for four weeks results in skin thinning and increased TEWL [2]. T-helper 1 (Th1) cells predominate in the skin lesions of chronic AD, whereas T-helper 2 (Th2) cytokine levels are often elevated in the skin lesions of acute AD. Thymus and activation-regulated chemokine (TARC), which is synthesized by keratinocytes, is a Th2-type chemokine and plays a pivotal role in the onset and progression of AD [3]. In addition, the expression of squamous cell carcinoma antigen (SCCA) 2, a serine protease inhibitor, is upregulated in response to Th2-type cytokines, particularly interleukin (IL)-4 and 13 [4]. TARC and SCCA2 are prominent inflammatory biomarkers intrinsically associated with Th2-dominated immune responses. In AD, serum TARC and SCCA2 levels are indicative of disease severity and progression [5-7]. These biomarkers are key indicators of the effectiveness of AD treatment.

In addition, systemic therapies for AD, including oral Janus kinase (JAK) inhibitors and biologics, have shown effectiveness when used with traditional topical treatments. For example, dupilumab, which specifically targets the IL-4Rα receptor subunit of IL-4 and IL-13, reduces TEWL and consistently downregulates Th2 cytokines in both eczematous and non-eczematous skin [8]. Conversely, upadacitinib is an oral JAK inhibitor with JAK1 selectivity [9]. Upadacitinib is more effective than dupilumab against AD [10] and can improve skin barrier function and reduce Th2 cytokines. However, few studies have simultaneously evaluated these parameters in patients with AD treated with upadacitinib, particularly in pediatric populations. In this context, we present two pediatric patients with AD in whom changes in serum TARC and SCCA2 levels and TEWL were evaluated for four weeks after starting upadacitinib treatment.

## Case presentation

We retrospectively evaluated two children with severe AD by measuring TEWL using a Tewameter TM Hex device (Courage + Khazaka, Köln, Germany) and serum TARC and SCCA2 levels for four weeks after starting upadacitinib treatment. The study protocol was approved by the Ethics Review Committee of Jikei University School of Medicine (approval number 35-029:11650]), and the patients and their parents provided informed consent to participate in the study. This study complied with the Ethical Guidelines for Life Sciences and Medical Research Involving Human Subjects in Japan. The two patients and their parents were informed about the case report and provided written informed consent for the publication of their case details and the associated images.

The 11-year-old girl and 12-year-old boy evaluated in this study had persistent severe AD despite treatment by their primary care providers for six months to two years, respectively. We describe the clinical course of the two cases. The first case is that of an 11-year-old girl who presented with AD and had an eczema area and severity index (EASI) score of 36.3 at the initial consultation. She was treated with 0.12% betamethasone valerate ointment for facial eczema and was switched to maintenance therapy with 0.03% topical tacrolimus. Successful remission of her generalized eczema was achieved using 0.05% betamethasone butyrate propionate ointment. Her ongoing maintenance therapy includes an ointment containing 0.12% betamethasone valerate combined with 0.5% delgocitinib, a topical JAK inhibitor. Despite regularly using the antihistamine fexofenadine, remission was not maintained over six months of intensive treatment; therefore, oral upadacitinib was initiated when she reached 12 years of age.

The second case is that of a boy who presented at the age of 12 years with AD and had an EASI score of 29.4 at the initial consultation. His facial eczema was treated with 0.12% betamethasone valerate ointment, followed by maintenance therapy with 0.03% topical tacrolimus. Remission of the generalized eczema was achieved with a 0.05% betamethasone butyrate propionate ointment and maintained with an ointment containing 0.12% betamethasone valerate combined with 0.5% delgocitinib. Oral antihistamines, including desloratadine and fexofenadine, were prescribed in conjunction with these treatments. However, despite two years of intensive topical treatment, the eczema persisted, necessitating the initiation of upadacitinib therapy.

**Figure 1:**
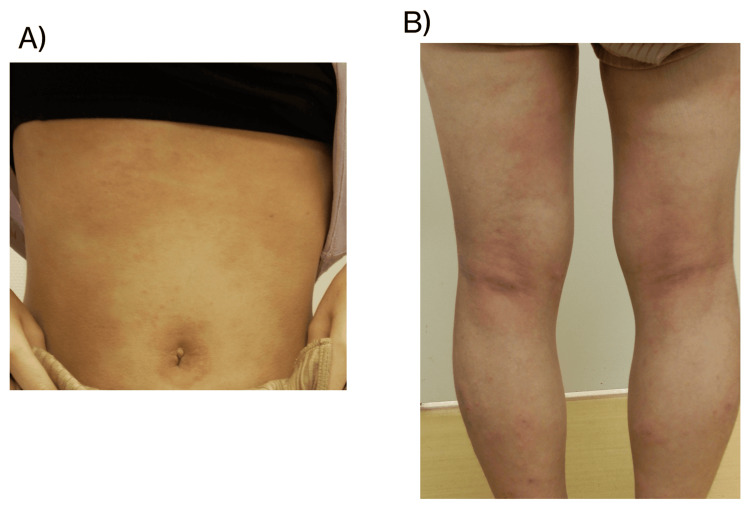
Dermatologic findings in a 12-year-old girl with severe AD before upadacitinib treatment (A) Abdominal lesions and (B) involvement of the flexural region of the lower leg. The patients had an EASI score of 34, serum TARC level of 19,180 pg/mL, and TEWL on the lateral side of the lower leg of 21.5 g/h/m² EASI: eczema area and severity index, TARC: thymus and activation-regulated chemokine, TEWL: transepidermal water loss, AD: atopic dermatitis

**Figure 2:**
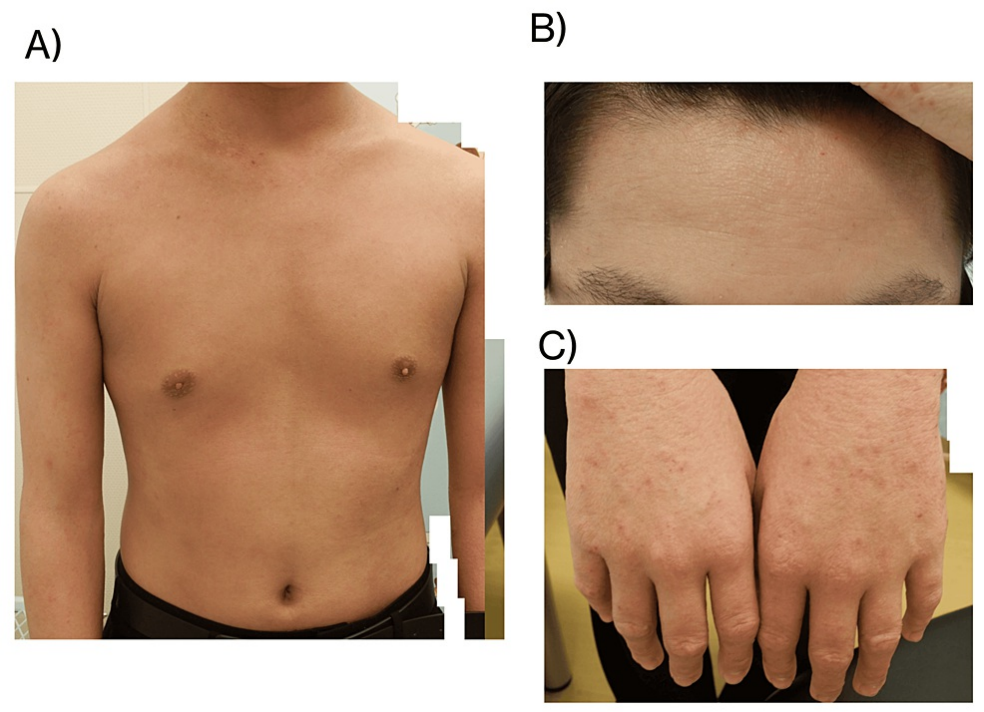
Dermatologic findings in a 14-year-old boy with severe AD before upadacitinib treatment Lesions on the (A) trunk, (B) forehead, and (C) dorsum of the hands. The patient had an EASI score of 27.6, serum TARC level of 7,270 pg/mL, and TEWL on the lateral aspect of the forearm of 25.22 g/h/m² EASI: eczema area and severity index, TARC: thymus and activation-regulated chemokine, TEWL: transepidermal water loss, AD: atopic dermatitis

Figures 1-2 show skin lesions immediately prior to the initiation of treatment with oral upadacitinib in the girl and boy, respectively.

**Figure 3:**
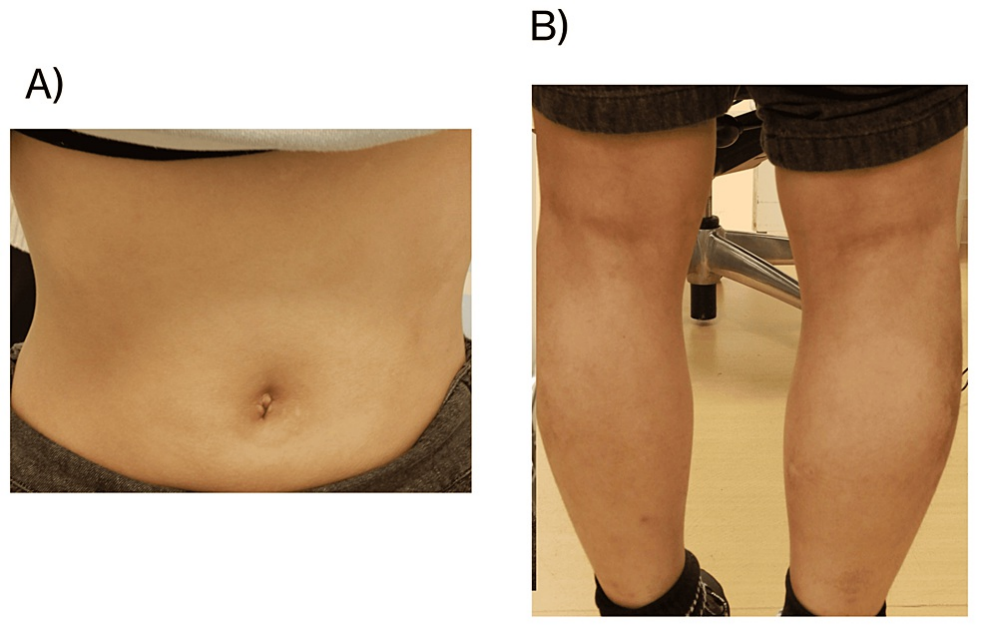
Dermatologic findings in the 12-year-old girl shown in Figure 1 after four weeks of upadacitinib treatment EASI score was 1.7, serum TARC level was 1.445 pg/mL, and TEWL on the lateral aspect of the lower leg was 14.45 g/h/m² EASI: eczema area and severity index, TARC: thymus and activation-regulated chemokine, TEWL: transepidermal water loss

**Figure 4:**
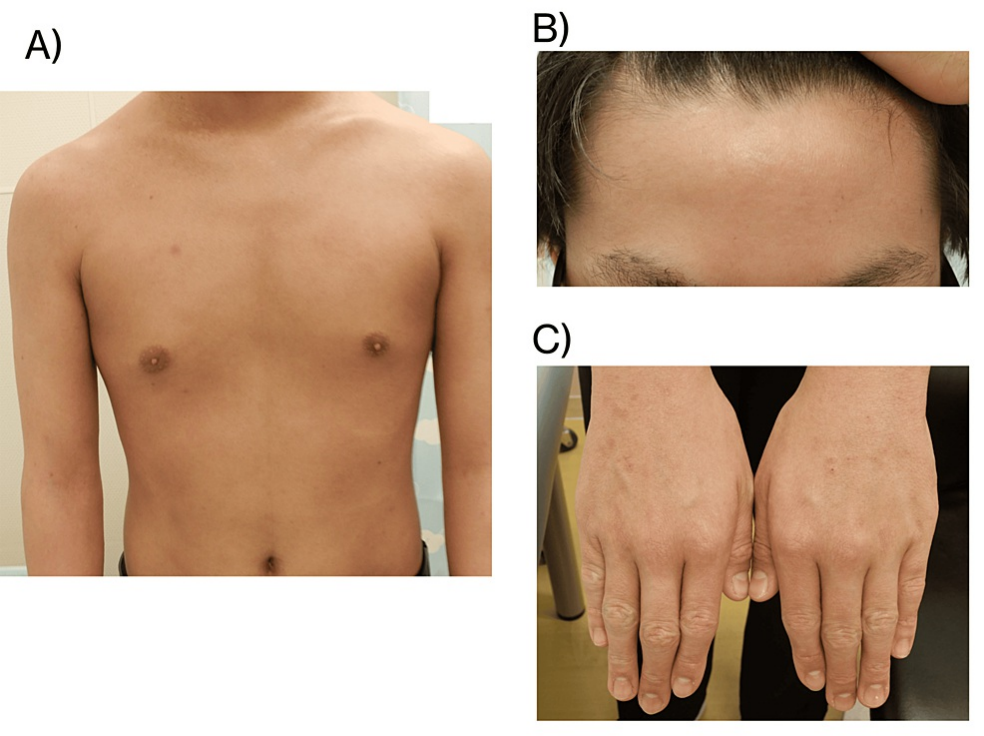
Dermatologic findings in the 14-year-old boy shown in Figure 2 after four weeks of upadacitinib treatment EASI score was 0.6, serum TARC level was 3.739 pg/mL, and TEWL on the lateral aspect of the forearm was 19.13 g/h/m² EASI: eczema area and severity index, TARC: thymus and activation-regulated chemokine, TEWL: transepidermal water loss

Therefore, they were treated with 15 mg of oral upadacitinib daily. The EASI, investigator's global assessment (IGA), and patient-oriented eczema measure (POEM) scores improved after four weeks of upadacitinib treatment. Both patients were severely symptomatic and remained on topical steroids, tacrolimus, and delgocitinib. Skin improvement was evident within the first week of treatment and was maintained after four weeks (Figures 3-4).

In addition, their blood eosinophil counts and serum TARC, SCCA2, and lactate dehydrogenase (LDH) levels decreased after four weeks of upadacitinib treatment. After four weeks of treatment, TEWL measurements of the palmar forearm, lateral forearm, and lateral aspect of the lower leg were markedly improved compared with their values in the first week of treatment (Table 1).

**Table 1:** Patients’ characteristics and clinical course ^a^ An 11-year-old girl with several AD, ^b^ A 12-year-old boy with severe AD EASI: eczema area and severity index, IGA: investigator’s global assessment, IgE: immunoglobulin E, LDH: lactate dehydrogenase, POEM: patient-oriented eczema measure, SCCA2: squamous cell carcinoma antigen 2, TARC: thymus and activation-regulated chemokine, TEWL: transepidermal water loss, AD: atopic dermatitis

Parameters	Baseline	1 week	2 or 3 weeks	4 weeks
Patient 1^a^				
EASI	34	12.8	5.1	1.7
IGA	4	2	2	1
POEM	14	3	4	1
Total IgE (IU/mL)	49,180	···	···	62,270
TARC (pg/mL)	19,180	···	···	1,445
SCCA2 (ng/mL)	60.4	···	···	0.5
LDH (IU/L)	417	···	···	164
Eosinophil count (/μL)	1,822	···	···	344
Temperature at TEWL measurement (°C)	25.4	25.3	26.5	24.8
Relative humidity at TEWL measurement (%)	32	34	34	29
Palmar forearm TEWL (g/h/m²)	21.9	18.68	13.64	11.37
Lateral forearm TEWL (g/h/m²)	90.07	25.61	14.87	13.28
Lateral lower leg TEWL (g/h/m²)	21.5	16.74	11.47	14.45
Patient 2^b^				
EASI	27.6	4.2	1.8	0.6
IGA	3	1	1	0
POEM	20	7	6	5
Total IgE (IU/mL)	3,381	···	···	4,205
TARC (pg/mL)	7,270	···	···	3,739
SCCA2 (ng/mL)	14.8	···	···	3.3
LDH (IU/L)	316	···	···	246
Eosinophil count (/μL)	967	···	···	533
Temperature at TEWL measurement (°C)	26.9	26.6	27.4	25.7
Relative humidity at TEWL measurement (%)	30	29	37	25
Palmar forearm TEWL (g/h/m²)	32.23	22.92	17.85	15.16
Lateral forearm TEWL (g/h/m²)	25.22	21.97	19.57	19.13
Lateral lower leg TEWL (g/h/m²)	27.08	19.27	20.49	17.51

The temperature during TEWL measurements varied between 24.8°C and 27.4°C, with relative humidity ranging from 25% to 37% (Table 1). Throughout the four-week treatment period, upadacitinib did not manifest any significant side effects necessitating treatment discontinuation.

## Discussion

By selectively inhibiting JAK and directly targeting various cytokine receptors, oral JAK inhibitors have shown therapeutic benefits in inflammatory diseases, including AD. Upadacitinib is particularly useful owing to its JAK1 specificity [11].

In this study, two patients showed inadequate improvement of skin-related symptoms even after intensive topical treatment prior to the initiation of upadacitinib therapy. The patients, who had no underlying psychiatric conditions, including autism, demonstrated good adherence to the prescribed topical drug therapy, as confirmed by their primary care physicians. However, it is important to recognize that patient adherence as reported during clinical consultations with healthcare professionals may not consistently reflect actual adherence to prescribed therapeutic protocols [12]. Thus, the possibility that the lack of significant improvement in patients' conditions may be due to suboptimal adherence to treatment protocols should not be overlooked. However, a review of the therapeutic efficacy of intensive treatments for AD showed that of 123 children with severe AD, only 107 achieved remission after undergoing a detailed treatment regimen that included topical steroids [13]. We attribute the lack of remission despite rigorous topical therapy to the severity of the disease while emphasizing the importance of treatment adherence.

Extensive clinical trials with biologics such as dupilumab, which targets the IL-4Rα receptor subunit of IL-4 and IL-13, have shown that they improve skin barrier function and cytokinesis [14]. Although upadacitinib theoretically suppresses the cytokines IL-4 and IL-13, potentially improving barrier function and reducing Th2 cytokine levels, empirical evidence is limited.

Recently, Hagino et al. [15] reported on a 12-week follow-up study of 31 patients aged 12 years and older with moderate to severe AD treated with upadacitinib. IgE, TARC levels, and blood eosinophil counts decreased.

Nevertheless, an increase in total IgE levels was observed in the two cases studied, indicating that the four-week treatment period with upadacitinib may not have been sufficient to achieve a significant reduction in total IgE levels. Specifically, the patient in Case 1 had a total IgE level of 18,588 IU/mL six months prior to starting upadacitinib treatment, while the patient in Case 2 had a total IgE level of 1,196 IU/mL two years prior to starting treatment. These results suggest that total IgE levels were elevated until the start of upadacitinib therapy, which may account for the observed increase in total IgE levels despite a decrease in serum TARC levels. Prior to the administration of upadacitinib, delgocitinib, a topical JAK inhibitor, was used. Although a JAK inhibitor, delgocitinib has minimal systemic absorption, resulting in extremely low blood concentrations [16]. Consequently, the influence of upadacitinib administration was deemed minimal. In addition, Hagino et al. [15] observed a reduction in total IgE levels after four weeks of upadacitinib treatment. However, this reduction was modest compared to the changes observed in serum TARC levels. In addition, only three of the patients were younger than 18 years of age, and important biomarkers, including SCCA2 levels and skin barrier function, were not assessed. Mizuno et al.’s study on serum TARC and serum IgE levels after systemic treatment with dupilumab for AD showed that serum TARC levels were more indicative of therapeutic progression in AD than serum IgE levels [17]. These results suggest that assessing the efficacy of upadacitinib in the treatment of adolescent AD based solely on total IgE levels may be inappropriate. Serum TARC and SCCA2 levels may provide a more accurate indication of treatment response. However, future studies should include long-term evaluations of upadacitinib and Th2 biomarkers to further substantiate these findings.

In our study, upadacitinib not only improved Th2-associated inflammatory markers but also improved skin barrier function within the first four weeks of treatment. Specifically, TEWL measurements of the palmar forearm, lateral forearm, and lateral lower leg improved within the first week and continued to improve during the first four weeks of treatment. An important consideration is that TEWL is influenced by external variables such as temperature and humidity [18]. However, in the two cases evaluated in this study, fluctuations in temperature and relative humidity were minimal, and their influence on TEWL was considered insignificant due to the relatively short study period (four weeks).

In addition, inflammatory biomarkers, including serum TARC, SCCA2, LDH levels, and eosinophil counts, showed improvement at four weeks. However, in our two cases, no decrease in total IgE levels was observed. This may be due to the relatively lower power of total IgE to reflect disease activity compared to other biomarkers. Upadactinib appears to strengthen the skin barrier function and attenuate Th2-associated cytokines during the initial period of use. However, no studies have evaluated the long-term effects of oral JAK inhibitors, including upadacitinib, on Th2 cytokines or skin barrier function. Thus, longitudinal studies are required to investigate these biomarkers with a longer follow-up period.

Skin barrier function parameters, such as stratum corneum water content or TEWL, are useful for determining the therapeutic effect of dupilumab [19]. Therefore, it is important to monitor clinical parameters, skin barrier integrity, and Th2 cytokine concentrations during oral JAK inhibitor therapy, with a focus on long-term assessment. Our study is limited by its small sample size and short duration of follow-up.

## Conclusions

We evaluated the serum levels of TARC and SCCA2, which are indicators of Th2-mediated inflammation, and TEWL, a measure of skin barrier function, during four weeks of treatment with the oral JAK inhibitor upadacitinib. To our knowledge, no clinical trials have simultaneously evaluated these biomarkers during upadacitinib treatment. In patients with AD treated with oral JAK inhibitors, concurrent assessment of Th2-associated inflammatory markers and skin barrier function is useful for monitoring the effectiveness of treatment. Long-term studies of larger numbers of patients are warranted.
